# Efficacy and Safety of Janus Kinase Inhibitors in Polymyalgia Rheumatica: A Systematic Review

**DOI:** 10.7759/cureus.118872

**Published:** 2026-10-31

**Authors:** Yumna E Elsayed, Jana Hegazi, Reem Elemam, Sumaya Elashmawy, Aya Harish, Yong Jia Lee, Meryem Karamoglu, Ahmad Harish, Mohamed E Elsayed

**Affiliations:** 1 Accident and Emergency, United Lincolnshire Hospitals NHS Trust, Boston, GBR; 2 Cardiology, University Hospitals of Leicester NHS Trust, Leicester, GBR; 3 Pediatric Emergency Medicine, University Hospitals of Leicester NHS Trust, Leicester, GBR; 4 Pediatrics, Blackpool Victoria Hospital, Blackpool, GBR; 5 Respiratory Medicine, University Hospitals of Leicester NHS Trust, Leicester, GBR; 6 Cardiology, Manchester University NHS Foundation Trust, Manchester, GBR; 7 Medicine, Newcastle University Medicine Malaysia, Johor Bahru, MYS; 8 Medicine, Newcastle University, Newcastle upon Tyne, GBR; 9 Palliative Care, University Hospitals of Leicester NHS Trust, Leicester, GBR

**Keywords:** baricitinib, glucocorticoid-sparing, janus kinase inhibitors, polymyalgia rheumatica, systematic review, tofacitinib

## Abstract

Polymyalgia rheumatica (PMR) is a common inflammatory disease of older adults; glucocorticoids remain the mainstay of treatment despite substantial cumulative toxicity. Janus kinase (JAK) inhibitors block cytokine signalling implicated in PMR, but their role has not been systematically appraised. We conducted a systematic review, prospectively registered on the International Prospective Register of Systematic Reviews (PROSPERO; CRD420261364002) and reported according to the Preferred Reporting Items for Systematic Reviews and Meta-Analyses (PRISMA) 2020 statement and the Synthesis Without Meta-analysis (SWiM) guideline. Embase, Medical Literature Analysis and Retrieval System Online (MEDLINE), the Cochrane Library, and PubMed were searched from inception to 18 May 2026 without language or date restrictions. Eligible studies reported original efficacy or safety data for a JAK inhibitor in adults with PMR diagnosed using an established framework. Risk of bias was assessed with design-matched tools, findings were synthesised narratively by clinical comparison, and certainty was rated with the Grading of Recommendations Assessment, Development and Evaluation (GRADE) approach separately for each comparison. Four studies (154 participants randomised or enrolled, 86 receiving a JAK inhibitor) contributed to the primary synthesis, and a mixed PMR and giant cell arteritis (GCA) case series was retained as context. In the only double-blind placebo-controlled trial, baricitinib increased the proportion of patients achieving low disease activity without oral glucocorticoids at week 12 (77.8% vs 13.3%; relative risk (RR) 5.8, 95% confidence interval (CI) 3.2-10.6). An open-label trial reported tofacitinib to be non-inferior to prednisone in a complete analysis in which every participant in both arms responded; on recalculation, this conclusion held only at the margin boundary and was not robust to missing data. A retrospective cohort and a single-arm study suggested glucocorticoid sparing but could not separate a drug effect from confounding or concurrent tapering. Certainty was low for the randomised comparisons and very low otherwise, and safety data were too sparse to characterise uncommon harms. Baricitinib may increase glucocorticoid-free low disease activity in recent-onset PMR (low certainty), and evidence for tofacitinib is of low to very low certainty. These preliminary findings do not establish efficacy for JAK inhibitors as a class, support routine use, or identify a preferred patient population; adequately powered, blinded trials are needed.

## Introduction and background

Polymyalgia rheumatica (PMR) is an inflammatory disease of the shoulder and pelvic girdles affecting adults over 50 years [1]. Glucocorticoids remain the mainstay of treatment and usually produce rapid improvement, but prolonged exposure is associated with clinically important morbidity, particularly in older adults [2,3]. Relapse during glucocorticoid tapering is common, and some patients remain exposed to glucocorticoids for prolonged periods [4]. Methotrexate is recommended as a glucocorticoid-sparing option for patients at high risk of relapse or prolonged glucocorticoid therapy [2], and interleukin-6 (IL-6) receptor inhibitors have since been evaluated in randomised trials; these alternatives are compared with Janus kinase (JAK) inhibition in the Discussion section.

JAK inhibitors block JAK-signal transducer and activator of transcription signalling, through which several cytokines implicated in PMR pathogenesis, including IL-6, signal [5]. JAK inhibitors are established treatments for several inflammatory diseases and have shown preliminary efficacy in giant cell arteritis (GCA) and other related conditions [5,6]. Whether JAK inhibition can reduce or avoid glucocorticoid exposure in PMR, and how its safety should be characterised in this older population, remains uncertain.

The evidence base has grown quickly. The first randomised trials of baricitinib and tofacitinib in PMR were published between 2023 and 2025 [7,8], and recent narrative reviews have discussed the promise of JAK inhibition [9]. Narrative reviews, however, do not apply a prespecified search, formal risk-of-bias assessment, or graded certainty of evidence, and they tend to read trials with different comparators as support for a single class effect. To our knowledge, at the time of our search, this is the first prospectively registered systematic review of JAK inhibitors in PMR to rate certainty separately for each clinical comparison: JAK inhibitor versus placebo, versus glucocorticoids, versus conventional disease-modifying antirheumatic drugs (DMARDs), and as uncontrolled add-on therapy. The distinction matters clinically, because "glucocorticoid-sparing" describes several different treatment strategies in this literature.

We, therefore, conducted a systematic review of comparative and non-comparative evidence evaluating JAK inhibitors for PMR, explicitly separating the evidence by clinical comparison and study design to avoid treating all JAK inhibitor studies as estimates of a common treatment effect.

## Review

Methods

Protocol and Reporting

The review was prospectively registered on the International Prospective Register of Systematic Reviews (PROSPERO; CRD420261364002). Reporting follows the Preferred Reporting Items for Systematic Reviews and Meta-Analyses (PRISMA) 2020 statement [10] and the Synthesis Without Meta-analysis (SWiM) reporting guideline [11]. The registered protocol planned a meta-analysis where appropriate; this was not performed, for the reasons given under the Effect Measures and Synthesis section. The recalculation of effect estimates and confidence intervals (CIs) for BACHELOR [7] and EAST PMR [8] was performed post hoc and was not specified in the registration. Certainty of evidence was assessed separately for clinically coherent comparisons rather than applying a common estimate across heterogeneous intervention and comparator strategies. The completed PRISMA 2020 checklist is provided in Appendix A.

Eligibility Criteria

Studies were eligible if they reported original efficacy and/or safety data for a JAK inhibitor administered to adults with PMR. Randomised controlled trials, non-randomised comparative studies, prospective single-arm studies, and retrospective case series were eligible when the diagnostic approach was sufficiently described and compatible with an established PMR diagnostic or classification framework.

The 2012 American College of Rheumatology/European Alliance of Associations for Rheumatology (ACR/EULAR) classification criteria [12] were the reference framework, because they are reproducible and were used in the principal randomised evidence. They were not an eligibility restriction. The criteria were designed to create consistent research cohorts rather than to serve as a diagnostic standard, and other established approaches remain legitimate in clinical and observational research. We, therefore, recorded the framework each study used and judged case by case whether its population was comparable to the primary evidence base. EAST PMR [8], for example, accepted either the 1982 Chuang criteria [13] or the 2012 ACR/EULAR criteria [12] and was included in the primary synthesis. Camellino et al. [14] used the Bird criteria [15] in a mixed PMR/GCA population; this report was retained as supplementary contextual evidence rather than combined with the primary efficacy estimates.

Studies reporting only a new measurement instrument, duplicate or companion reports from an included cohort, and studies reporting outcomes unrelated to efficacy or safety were excluded.

Information Sources and Search Strategy

Embase (Ovid), Medical Literature Analysis and Retrieval System Online (MEDLINE, Ovid), the Cochrane Library (Cochrane Controlled Register of Trials (CENTRAL) and Cochrane Reviews), and PubMed were searched from inception to 18 May 2026, without language or publication-date restrictions. MEDLINE and PubMed were both searched because they are not identical retrieval systems: PubMed includes records not yet indexed in MEDLINE at the time of retrieval. Searching both maximised sensitivity in this emerging literature, and duplicates were removed before screening. Trial registries and the reference lists of included studies were not searched separately; CENTRAL includes records from ClinicalTrials.gov and the World Health Organization International Clinical Trials Registry Platform.

Each search combined three concepts: population, intervention (JAK inhibitors as a drug class and by 11 named agents), and outcome (efficacy, safety, adverse events). Comparator terms were not used. Complete database-specific strategies, including exact query syntax and the Cochrane run log with line-by-line hit counts, are provided in Appendix B.

Study Selection and Data Collection

Two reviewers independently screened records at title/abstract and full-text stages, with disagreements resolved by a third reviewer. Data were extracted independently using a standardised, piloted form covering study design, population, intervention, comparator, participant numbers at randomisation or enrolment, completion, and analysis, efficacy and safety outcomes, and risk-of-bias judgements. Safety data extracted included adverse events, serious adverse events, deaths, infections including herpes zoster, malignancy, major cardiovascular and thromboembolic events, laboratory abnormalities, treatment discontinuations, and duration of follow-up; outcomes not reported were distinguished from outcomes assessed but not observed.

Risk of Bias

Risk of bias was assessed with a tool matched to study design: the Cochrane Risk of Bias 2 (RoB 2) tool for randomised trials [16], the Risk Of Bias In Non-randomised Studies of Interventions (ROBINS-I) tool for the non-randomised comparative cohort [17], and the National Heart, Lung, and Blood Institute (NHLBI) Quality Assessment Tool for Before-After Studies With No Control Group for the single-arm study [18]. A second reviewer independently checked all judgements.

Effect Measures and Synthesis

For comparative efficacy outcomes, effect measures were reported as presented in the original studies. For BACHELOR [7], the primary endpoint was summarised using the relative risk (RR) and 95% CI reported by the investigators, derived from a generalised linear model with a binomial distribution and log link accounting for clustering by study centre; the unadjusted RR was also recalculated post hoc from the reported event counts using the log (Katz) method. EAST PMR [8] was treated as a non-inferiority comparison rather than converted into a superiority estimate. Because the confidence limits were not reported, the review authors recalculated, post hoc, the 95% CI for the difference in response proportions from the published event counts using the Newcombe hybrid score method, in the completer population and in all randomised participants under alternative assumptions about missing outcomes. For the single-arm study, proportions and 95% CIs were reported where available.

Meta-analysis was not performed because the four studies differed materially in JAK inhibitor, dose, concomitant glucocorticoid exposure, comparator, outcome definition, follow-up, and design. The synthesis was organised around four clinically coherent comparisons: JAK inhibitor versus placebo, JAK inhibitor versus glucocorticoid, JAK inhibitor versus conventional DMARD therapy, and uncontrolled glucocorticoid-sparing add-on therapy. This follows SWiM principles and avoids implying a common underlying treatment effect where the studies address different clinical questions.

Certainty of Evidence

The Grading of Recommendations Assessment, Development and Evaluation (GRADE) approach [19] was applied separately to each clinically coherent comparison rather than to composite outcomes combining heterogeneous designs and comparators. Randomised evidence started at high certainty and was downgraded for study limitations, inconsistency, indirectness, imprecision, and publication bias where appropriate. Non-randomised and single-arm evidence was downgraded substantially where confounding, lack of a comparator, or other design limitations impaired causal interpretation. With only four studies, funnel plots and Egger's test were not considered informative. Each domain was judged separately for each comparison: risk-of-bias judgements were taken directly from the domain-level assessments, inconsistency was not applicable where a comparison rested on a single study, and publication bias could not be assessed formally, so certainty was not downgraded for it.

Results

Study Selection

The search identified 241 records (Embase 184, MEDLINE 11, Cochrane Library 21, PubMed 25). After removal of 55 duplicates, 186 records were screened, and 180 were excluded at title/abstract review. Six reports were assessed at full text. One, a companion report from the EAST PMR cohort [8] describing a novel functional measure [20], was excluded to avoid double-counting participants. Five reports, corresponding to five unique studies, met the eligibility framework; each study was described in a single report. Four studies, with 154 participants randomised or enrolled (86 receiving a JAK inhibitor; 144 in the efficacy analyses), contributed to the primary efficacy synthesis. The study by Camellino et al. [14] (six patients, four with a PMR component) was retained as supplementary contextual evidence because of its mixed PMR/GCA population and alternative diagnostic framework and is not included in these totals (Figure 1).

**Figure 1:**
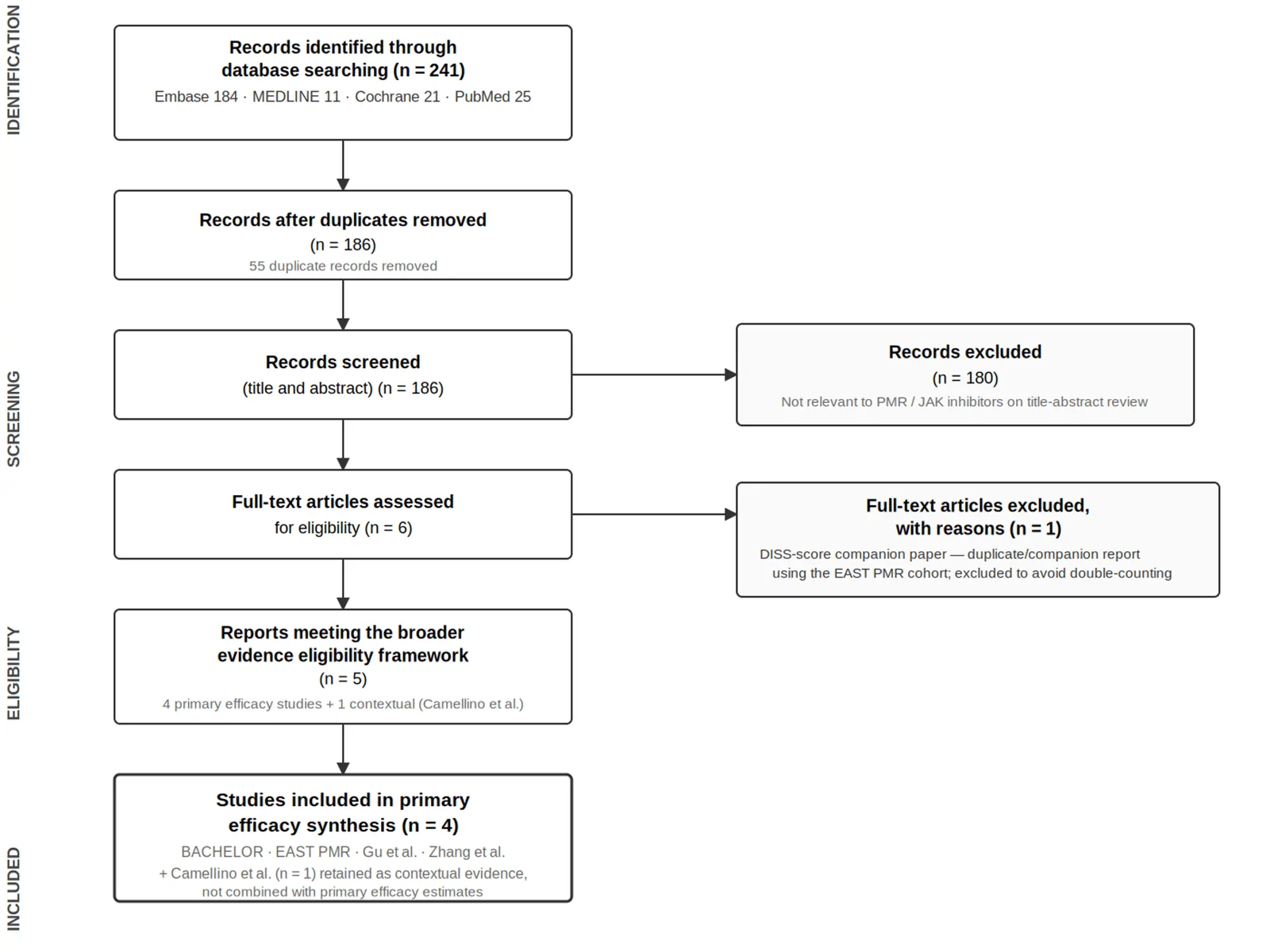
PRISMA 2020 flow diagram of study identification and selection Five reports corresponded to five unique studies (one report per study). Studies included in the primary synthesis: BACHELOR [7], EAST PMR [8], Gu et al. [21], and Zhang et al. [22]. Retained as contextual evidence: Camellino et al. [14]. Excluded at full text: DISS score companion report [20] of the EAST PMR cohort [8]. PRISMA: Preferred Reporting Items for Systematic Reviews and Meta-Analyses; MEDLINE: Medical Literature Analysis and Retrieval System Online; PMR: polymyalgia rheumatica; JAK: Janus kinase; DISS: difficulty in squatting and standing up

Characteristics of the Included Studies

The included evidence comprised one double-blind placebo-controlled randomised trial of baricitinib [7], one open-label randomised non-inferiority trial of tofacitinib versus glucocorticoids [8], one retrospective matched cohort of tofacitinib versus methotrexate-based therapy [21], and one single-arm phase 2 study of tofacitinib given with a prednisone taper [22]. Their characteristics are summarised in Table 1.

**Table 1:** Characteristics of the four studies included in the primary synthesis Where two numbers are separated by "vs", the first refers to the JAK inhibitor group and the second to the comparator group; the single-arm study reports one group only. Efficacy and safety analysis populations are shown separately where they differed. RCT: randomised controlled trial; GC: glucocorticoid; DMARD: disease-modifying antirheumatic drug; CRP: C-reactive protein; PMR-AS: polymyalgia rheumatica activity score; ACR/EULAR: American College of Rheumatology/European Alliance of Associations for Rheumatology; GCA: giant cell arteritis; NSAIDs: non-steroidal anti-inflammatory drugs; JAK: Janus kinase; PMR: polymyalgia rheumatica; vs: versus

Study (design)	JAK inhibitor	Comparator	Population	Randomised/enrolled, n (JAK inhibitor vs comparator)	Analysed, n (JAK inhibitor vs comparator)	Concomitant GC/DMARD
Saraux et al. [7] (BACHELOR; double-blind RCT)	Baricitinib 4 mg/day weeks 0-12, then 2 mg/day weeks 12-24	Placebo	Recent-onset (<6 months), GC-naive, CRP PMR-AS >17; 2012 ACR/EULAR criteria [12]	18 vs 16	Efficacy 18 vs 15; safety 18 vs 16	No oral GC initially; rescue oral GC and subdeltoid GC injections permitted
Ma et al. [8] (EAST PMR; open-label non-inferiority RCT)	Tofacitinib 5 mg twice daily for 24 weeks	Prednisone 15 mg/day, tapered	Newly diagnosed, treatment-naive, PMR-AS >10; 1982 Chuang criteria [13] or 2012 ACR/EULAR criteria [12]	39 vs 37	35 vs 32 completers	Tofacitinib monotherapy (NSAIDs permitted for the first 2 weeks); control received prednisone
Gu et al. [21] (retrospective matched cohort)	Tofacitinib 5 mg twice daily	Methotrexate plus GC	All female; 2012 ACR/EULAR criteria [12]; isolated PMR except one with GCA; two previously treated with DMARD plus GC	15 vs 15 (18 JAK inhibitor-treated identified, three excluded)	15 vs 15 (13 vs 15 at six months)	14/15 also received methotrexate and 11/15 GC; all controls received methotrexate plus GC
Zhang et al. [22] (single-arm phase 2)	Tofacitinib 5 mg twice daily (10 mg/day)	None	PMR-AS >17; 11 newly diagnosed, three relapsing	14	14	Prednisone 15 mg/day with protocolised taper

Risk of Bias

BACHELOR [7] was judged to have some concerns, driven mainly by differential discontinuation and the potential for partial unmasking through laboratory trends. EAST PMR [8] was judged at high risk of bias overall, driven by unblinded assessment of the polymyalgia rheumatica activity score (PMR-AS), which contains subjective components (Domain 4), with some concerns for deviations from intended interventions (completer-only analysis) and missing outcome data (Table 2). Gu et al. [21] had serious risk of confounding because treatment allocation was non-random and baseline glucocorticoid exposure differed between groups (9.47 vs 15.33 mg/day). Zhang et al. [22] was judged fair on the NHLBI tool, with lack of blinding and a single pre-treatment baseline measurement limiting causal inference.

**Table 2:** Risk-of-bias assessment of the randomised controlled trials (RoB 2) Overall judgement follows the RoB 2 algorithm: high risk in any domain leads to an overall judgement of high risk RoB 2: Cochrane Risk of Bias 2 tool; D: domain; CRP: C-reactive protein; PMR: polymyalgia rheumatica; PMR-AS: polymyalgia rheumatica activity score.

Study	D1: Randomisation process	D2: Deviations from intended interventions	D3: Missing outcome data	D4: Measurement of the outcome	D5: Selection of the reported result	Overall
Saraux et al. [7] (BACHELOR)	Low: 1:1 allocation stratified by hospital in random blocks of two, four, and six, assigned centrally through the electronic case report form; identical placebo; participants, investigators, and outcome assessors masked	Low: double-blind; rescue glucocorticoid use was captured within the primary endpoint definition	Some concerns: differential treatment discontinuation (six vs 11 participants); one placebo participant withdrew before treatment and was excluded from the efficacy analysis; no imputation	Some concerns: possible partial unmasking through laboratory trends, as CRP contributes to the outcome measure	Low: prospectively registered (NCT04027101) with a prespecified primary endpoint	Some concerns
Ma et al. [8] (EAST PMR)	Some concerns: random numbers generated online with envelope allocation; concealment of the envelopes not described	Some concerns: open-label; few deviations (three participants self-reduced the tofacitinib dose); efficacy analysed in completers (67/76) rather than all randomised participants, with small and balanced exclusions	Some concerns: week 24 outcome data for 67/76 (88%); losses balanced (4/39 vs 5/37) and reported as responders before loss (to week 12 and week 16, respectively); no imputation or sensitivity analysis	High: unblinded assessment of an outcome with subjective components (PMR-AS)	Low: prospectively registered (ChiCTR2000038253; registered 15 September 2020, first enrolment 20 September 2020) with prespecified primary outcome and non-inferiority margin; primary outcome reported at both prespecified time points	High

Domain-level judgements are presented by study design: Table 2 for the randomised trials (RoB 2), Table 3 for the non-randomised comparative cohort (ROBINS-I), and Table 4 for the single-arm study (NHLBI tool).

**Table 3:** Risk-of-bias assessment of the non-randomised comparative cohort (ROBINS-I), Gu et al.. Study assessed: Gu et al. [21]. ROBINS-I: Risk Of Bias In Non-randomised Studies of Interventions

Domain	Judgement	Support for judgement
Bias due to confounding	Serious	Treatment allocated by clinician choice; groups matched on age and sex only; baseline prednisone dose differed (9.47 vs 15.33 mg/day); no statistical adjustment
Bias in selection of participants	Moderate	Post hoc exclusion of three of 18 tofacitinib-treated patients and nine of 24 comparator patients (tripterygium use)
Bias in classification of interventions	Low	Treatment groups defined from prescribing records
Bias due to deviations from intended interventions	Moderate	Fourteen of 15 tofacitinib-treated patients also received methotrexate, and 11 received concomitant glucocorticoid
Bias due to missing data	Moderate	Two patients excluded for missing laboratory data; 13 of 15 tofacitinib-treated patients assessed at six months
Bias in measurement of outcomes	Moderate	Outcomes extracted retrospectively from records; glucocorticoid dosing decided by clinicians aware of treatment
Bias in selection of the reported result	Moderate	No published protocol; multiple outcomes and time points reported with p-values only
Overall	Serious	Driven by confounding by indication

**Table 4:** Quality assessment of Zhang et al., the single-arm study (NHLBI Quality Assessment Tool for Before-After Studies With No Control Group) Study assessed: Zhang et al. [22] NHLBI: National Heart, Lung, and Blood Institute

Item	Criterion	Response
1	Was the study question or objective clearly stated?	Yes
2	Were eligibility/selection criteria for the study population prespecified and clearly described?	Yes
3	Were the participants representative of those who would be eligible for the intervention in the general or clinical population of interest?	Cannot determine
4	Were all eligible participants that met the prespecified entry criteria enrolled?	Not reported
5	Was the sample size sufficiently large to provide confidence in the findings?	Yes (Simon two-stage design)
6	Was the intervention clearly described and delivered consistently across the study population?	Yes
7	Were the outcome measures prespecified, clearly defined, valid, reliable, and assessed consistently across all study participants?	Yes
8	Were the people assessing the outcomes blinded to the participants' exposures/interventions?	No
9	Was the loss to follow-up after baseline 20% or less? Were those lost to follow-up accounted for in the analysis?	Yes
10	Did the statistical methods examine changes in outcome measures from before to after the intervention? Were statistical tests done that provided p-values for the pre-to-post changes?	Yes
11	Were outcome measures of interest taken multiple times before the intervention and multiple times after the intervention?	No (single pre-treatment baseline)
12	If the intervention was conducted at a group level, did the statistical analysis take into account the use of individual-level data to determine effects at the group level?	Not applicable
	Overall quality rating	Fair

JAK Inhibitor Versus Placebo

BACHELOR [7] was the only double-blind placebo-controlled trial. Eighteen participants were randomised to baricitinib and 16 to placebo; one placebo participant withdrew before treatment and was excluded from the efficacy analysis. At week 12, 14/18 (77.8%) participants receiving baricitinib versus 2/15 (13.3%) receiving placebo achieved a C-reactive protein-based PMR-AS ≤10 without oral glucocorticoid use from week 1 to week 12 (RR 5.8, 95% CI 3.2-10.6, centre-adjusted as reported [7]; unadjusted RR from event counts 5.83, 95% CI 1.57-21.70). Treatment effects were observed early, and disease control was reported to be maintained during follow-up, but the sample was small. The trial therefore provides direct evidence for a specific strategy, baricitinib, in recent-onset, glucocorticoid-naive, high-activity PMR, not for JAK inhibitors as a class across all PMR populations.

JAK Inhibitor Versus Glucocorticoid

EAST PMR [8] randomised 39 participants to tofacitinib and 37 to glucocorticoids. Four tofacitinib and five glucocorticoid participants were lost to follow-up, and the 35 and 32 completers formed the primary efficacy population. All completers in both arms had a PMR-AS <10 at weeks 12 and 24. The trial prespecified a non-inferiority margin of 0.10 for the difference in response proportions. The investigators compared completers using Fisher's exact test and concluded that non-inferiority was established, stating that the CI for the difference was 0 and therefore within the 0.1 margin; this is a degenerate interval rather than a valid CI, and the conclusion cannot be verified from the reported analysis. Our recalculation from the published counts using the Newcombe method gave a difference of 0% (95% CI -9.9 % to 10.7%) in completers, with the lower limit within the −10% margin by only 0.1 percentage points. The investigators reported that the four tofacitinib and five glucocorticoid participants lost to follow-up had responded at weeks 8 to 12 and 8 to 16, respectively; including them, non-inferiority was supported at week 12 (95% CI −9.0% to 9.4%). At week 24, their outcomes are unknown, and non-inferiority was not established if they were counted as non-responders (95% CI −12.1% to 19.0%). The trial also stopped recruitment below its own sample size target of 47 per arm once the high response rate became apparent. EAST PMR [8] accepted either the 1982 Chuang [13] or 2012 ACR/EULAR criteria [12] without reporting the split, so applicability to a strictly 2012 ACR/EULAR-defined population [12] is uncertain.

This result requires substantial qualification. The efficacy analysis was restricted to completers rather than all randomised participants. Attrition was similar between arms, and the authors reported that participants lost to follow-up had responded before leaving, but their week 24 outcomes are unknown, and a completer-only analysis departs from the analysis populations recommended for non-inferiority trials. The study was open-label, the outcome includes subjective components, and a 100% response in both arms creates a ceiling effect. Non-inferiority is therefore the investigators' conclusion; our recalculation supports it at the margin boundary in completers and at week 12, but the week 24 result depends on assumptions about participants lost to follow-up. It does not establish equivalence or superiority, and it should not be read as evidence that tofacitinib is interchangeable with glucocorticoid therapy.

JAK Inhibitor Versus Conventional DMARD Therapy

The retrospective matched cohort by Gu et al. [21] compared 15 participants receiving tofacitinib with 15 age- and sex-matched participants receiving methotrexate plus glucocorticoids. Fourteen of 15 participants (93.3%) in the tofacitinib group also received methotrexate, limiting attribution to JAK inhibition alone. Mean prednisone dose was lower in the tofacitinib group at baseline (9.47 vs 15.33 mg/day), three months (4.08 vs 7.08 mg/day), and six months (1.84 vs 5.25 mg/day), with 13 tofacitinib-treated participants remaining at six months. Because glucocorticoid exposure already differed substantially at baseline, the findings are vulnerable to confounding by indication and cannot establish a causal glucocorticoid-sparing effect.

Uncontrolled Glucocorticoid-Sparing Add-On Therapy

The phase 2 single-arm study by Zhang et al. [22] enrolled 14 participants with high-activity PMR, including 11 newly diagnosed and three relapsing patients. Tofacitinib was given with a protocolised prednisone taper. Twelve of 14 participants (85.7%, 95% CI 57.2-98.2%) achieved sustained low disease activity with glucocorticoid independence by approximately weeks 20-24. Because there was no comparator and glucocorticoid tapering occurred concurrently, the study cannot separate a drug effect from the natural disease course, regression to the mean, or the effects of glucocorticoid treatment.

Safety

Safety outcomes are summarised in Table 5. Follow-up ranged from 24 weeks to 36 weeks on randomised comparison, with an extension to 48 weeks in Zhang et al. [22]. BACHELOR [7] recorded four serious adverse events with baricitinib and six with placebo, including one serious infection with baricitinib, and one baricitinib-treated participant was diagnosed with uterine cancer that the investigators assessed as unrelated to treatment; there were no deaths or major adverse cardiovascular or thromboembolic events. EAST PMR [8] reported no serious adverse events, deaths, cardiovascular or thromboembolic events, malignancies, or discontinuations due to adverse events; herpes zoster occurred in 2/39 tofacitinib-treated participants (5.1%), and none with glucocorticoids, and new hyperlipidaemia was less frequent with tofacitinib (4/39 vs 11/37). Among the 18 tofacitinib-treated patients in Gu et al. [21], one developed herpes zoster (5.6%), which required temporary interruption of tofacitinib, and one 91-year-old patient, excluded from the efficacy analysis, had recurrent infections; serious adverse events, deaths, and other harms were not reported. In Zhang et al. [22], 12 adverse events occurred in five of 14 participants (35.7%), all grade 1-2, most commonly upper respiratory tract infection (4/14); two participants (14.3%) developed positive T-SPOT tests for latent tuberculosis, and no cardiovascular events, malignancy, or herpes zoster were recorded.

**Table 5:** Safety outcomes reported in the included studies Where two values are given, the first refers to the JAK inhibitor group and the second to the comparator group. NR: not reported; 0: assessed and not observed; AE: adverse event; GC: glucocorticoid; MTX: methotrexate; URTI: upper respiratory tract infection; TB: tuberculosis; MACE: major adverse cardiovascular events; ALT: alanine aminotransferase; JAK: Janus kinase; vs: versus

Outcome	BACHELOR [7]	EAST PMR [8]	Gu et al. [21]	Zhang et al. [22]
Safety population	18 baricitinib vs 16 placebo	39 tofacitinib vs 37 GC	18 tofacitinib vs 15 MTX plus GC	14 (single arm)
Follow-up	36 weeks (24 weeks on treatment)	24 weeks	6 months	24 weeks plus extension to 48 weeks
Any adverse event	50 vs 48 events	Reported individually (see below)	NR (individual events only)	12 events in 5/14 (35.7%), all grade 1-2
Serious adverse events	4 vs 6 events	0 vs 0	NR	0 (all events grade 1-2)
Deaths	0 vs 0	0 vs 0	NR	NR
Infections	6/18 vs 6/16; 1 serious (baricitinib)	Urinary 2/39 vs 0/37; no URTI or TB	Recurrent infections 1/18 (aged 91) vs NR	URTI 4/14; urinary 1/14
Herpes zoster	NR in main report	2/39 vs 0/37 (mild)	1/18 vs NR	0
Positive TB screening	NR	0 (no TB)	NR	Positive T-SPOT 2/14
Malignancy	1 vs 0 (uterine cancer, judged unrelated)	0 vs 0	NR	0
MACE or thromboembolism	0 vs 0	0 vs 0	NR	0
Laboratory abnormalities	NR in main report	Hyperlipidaemia 4 vs 11; cytopenia 1 vs 2; liver 0 vs 2	NR	ALT increase 1; creatinine increase 1
Treatment discontinuation	6 vs 11 (rescue 3 vs 6; other reasons 3 vs 4)	0 vs 0 due to AE; lost to follow-up 4 vs 5	Interrupted for zoster 1/18; NR otherwise	0 by week 24; 3 stopped tofacitinib by week 48 (protocol taper)

These observations show that infections, including herpes zoster, occurred during JAK inhibitor treatment, but they do not establish an incidence rate or relative risk in PMR. With 86 JAK inhibitor-treated participants in the analysed populations and follow-up of 24 to 48 weeks, the data are far too limited to assess uncommon infections, malignancy, major adverse cardiovascular events, or venous thromboembolism. Safety conclusions should therefore be considered exploratory.

Certainty of Evidence

Certainty of evidence for each clinically coherent comparison is summarised in Table 6.

**Table 6:** GRADE certainty of evidence for each clinically coherent comparison, with reasons for downgrading Publication bias could not be assessed formally for any comparison and did not lead to downgrading. "Not further downgraded": certainty was already very low. ^a^Some concerns from differential discontinuation (6 vs 11 participants) and possible partial unmasking because C-reactive protein contributes to the outcome measure (Table 2). ^b^33 participants and 16 events; unadjusted 95% CI 1.57-21.70; sample below the optimal information size. ^c^High risk of bias in Domain 4: unblinded assessment of an outcome with subjective components (Table 2). ^d^Recalculated lower confidence limit (−9.9%) at the −10% non-inferiority margin; not robust to missing week 24 data; recruitment stopped below the planned sample size; ceiling effect from 100% response in both arms. ^e^Serious confounding by indication and baseline glucocorticoid imbalance (Table 3). ^f^Fourteen of 15 tofacitinib-treated patients also received methotrexate, so the comparison reflects add-on therapy. ^g^No comparator; fair quality on the NHLBI tool (Table 4). ^h^A concurrent glucocorticoid taper prevents attribution of the effect to tofacitinib.^ i^Non-randomised safety data and selective reporting of harms (Table 5). GRADE: Grading of Recommendations Assessment, Development and Evaluation; RCT: randomised controlled trial; GC: glucocorticoid; LDA: low disease activity; CI: confidence interval; NHLBI: National Heart, Lung, and Blood Institute; JAK: Janus kinase; PMR: polymyalgia rheumatica; vs: versus

Comparison/outcome	Reference	Evidence (starting certainty)	Risk of bias	Inconsistency	Indirectness	Imprecision	Certainty	Interpretation
BACHELOR: low disease activity without oral GC at week 12	[7]	One RCT; 33 participants in efficacy analysis (high)	Serious (−1)^a^	Not applicable (single study)	Not serious	Serious (−1)^b^	Low	Baricitinib may increase GC-free low disease activity in recent-onset, GC-naive PMR
EAST PMR: non-inferiority of tofacitinib vs GC	[8]	One open-label RCT; 76 randomised, 67 completers analysed (high)	Serious (−1)^c^	Not applicable (single study)	Not serious	Serious (−1)^d^	Low	Non-inferiority was the investigators' conclusion; reproducible only at the margin boundary and dependent on missing week 24 data
Gu et al.: glucocorticoid-sparing effect	[21]	One retrospective cohort; 30 participants (low)	Serious (−1)^e^	Not applicable (single study)	Serious (not further downgraded)^f^	Serious (not further downgraded)	Very low	Very uncertain; hypothesis-generating only
Zhang et al.: sustained LDA with GC independence	[22]	One single-arm study; 14 participants (low)	Serious (−1)^g^	Not applicable (single study)	Serious (not further downgraded)^h^	Serious (not further downgraded)	Very low	Very uncertain; the treatment effect cannot be isolated
Safety: herpes zoster and rare harms	[7,8,21,22]	Four studies; 86 JAK inhibitor-treated participants in the analysed populations (89 including three patients excluded from the efficacy analysis of Gu et al.) (low)	Serious (−1)^i^	Serious (heterogeneous reporting)	Serious (follow-up 24 to 48 weeks)	Very serious (few events)	Very low	No reliable PMR-specific estimate of rare or serious harms can be made

Discussion

Principal Findings

This review identified four studies of JAK inhibitors in PMR, each addressing a different clinical question. The evidence is therefore better understood as a set of small, heterogeneous comparisons than as a single evidence base supporting a class-wide estimate. The strongest direct evidence comes from BACHELOR [7], in which baricitinib may have improved the probability of achieving low disease activity without oral glucocorticoids compared with placebo in recent-onset, glucocorticoid-naive, high-activity PMR (low certainty). EAST PMR [8] reported tofacitinib non-inferior to glucocorticoids in a small, open-label completer analysis, a conclusion that we could reproduce only at the margin boundary and that was not robust to assumptions about missing data. The observational and single-arm studies provide lower-certainty, hypothesis-generating information.

The available evidence does not establish a single best-supported patient population for JAK inhibitors. The included studies enrolled distinct populations: recent-onset, glucocorticoid-naive, high-activity PMR in BACHELOR [7]; newly diagnosed, treatment-naive disease in EAST PMR [8]; a previously treated observational population in Gu et al. [21]; and mixed newly diagnosed and relapsing disease receiving concomitant prednisone in Zhang et al. [22]. These populations should not be conflated when interpreting the findings.

Interpretation of Glucocorticoid-Sparing Efficacy

The term "glucocorticoid-sparing" covers different treatment strategies in the included literature. BACHELOR [7] evaluated avoidance of oral glucocorticoids as an initial strategy, Zhang et al. [22] evaluated JAK inhibition added to a prednisone taper, Gu et al. [21] compared groups with different baseline glucocorticoid exposure, and EAST PMR [8] evaluated tofacitinib as an alternative to a glucocorticoid regimen. These are clinically distinct questions rather than variations on one comparison, which is why certainty was rated per comparison rather than for a pooled "glucocorticoid-sparing effect".

Camellino et al. [14], retained as contextual evidence only, report findings in the same direction. Six patients received baricitinib for refractory disease or during a tocilizumab shortage; four had a PMR component (two isolated PMR, two PMR with large-vessel GCA). In this subgroup, median PMR-AS fell from 23.3 at baseline to 1.1 at last follow-up; three of four patients achieved a PMR-AS below 7, and two discontinued glucocorticoids entirely while continuing baricitinib monotherapy. Two patients in the wider cohort reverted to tocilizumab after about three months, one because of returning large-vessel disease activity and one after pneumonia, the series' only reported baricitinib-attributable adverse event. Because the series used the Bird criteria [15] and combined isolated PMR with PMR-associated GCA, it cannot be pooled with the primary evidence. Its direction of effect, a substantial reduction in disease activity and glucocorticoid dose, is nevertheless consistent with BACHELOR [7], and its one adverse event leading to discontinuation is consistent with the infection signal discussed below.

Clinical Interpretation and Patient Selection

The current evidence is insufficient to support evidence-based selection of particular PMR patients for JAK inhibitor therapy. The study populations may generate hypotheses for future trials, but the studies are too small and heterogeneous to show whether efficacy differs by disease duration, prior glucocorticoid exposure, relapse status, baseline inflammatory burden, or concomitant therapy. Any use of JAK inhibitors in PMR should be considered within local regulatory status, established treatment recommendations, individual patient risk, and clinical trial evidence, rather than inferred from subgroup impressions in these small studies.

Safety

The available PMR-specific safety evidence is inadequate for estimating uncommon but clinically important harms. Herpes zoster was observed in a small number of tofacitinib-treated participants, but event counts were low, and reporting was heterogeneous. No major cardiovascular or thromboembolic events were observed, and the single malignancy (in BACHELOR) was assessed as unrelated to treatment; the two positive T-SPOT conversions in Zhang et al. [22] underline the need for tuberculosis screening and monitoring in older patients. However, the absence of events in small cohorts followed for up to 48 weeks should not be read as evidence that they do not occur. An increased risk of major adverse cardiovascular events and malignancy was identified with tofacitinib in rheumatoid arthritis [23], concentrated in patients aged 65 years or older with cardiovascular risk factors, and this population overlaps substantially with people affected by PMR. Longer and larger PMR-specific studies are required to characterise safety in this older population.

Comparison With Other Targeted Therapies

IL-6 receptor inhibition has been studied in both glucocorticoid-dependent [24] and new-onset PMR [25], and sarilumab has demonstrated efficacy in relapsing PMR [26]. Abatacept, tested in a design similar to BACHELOR [7] that avoided initial glucocorticoids entirely, did not reach statistical significance for its primary endpoint [27], and methotrexate has shown inconsistent glucocorticoid-sparing effects across trials [28]. These studies provide useful context, but cross-trial comparisons should be interpreted cautiously because populations, outcome definitions, background glucocorticoid regimens, and designs differ. BACHELOR [7] is best described as evidence from a placebo-controlled JAK inhibitor trial, not as proof that JAK inhibition is more effective than other targeted mechanisms.

Limitations

The principal limitations arise from the evidence base. Only four studies were eligible, and two were randomised: the active-comparator trial was open-label and analysed completers only, the non-randomised comparison was confounded by indication, and the single-arm study had no comparator. Small samples and short follow-up constrain causal inference and safety assessment. Diagnostic frameworks also varied between studies. We handled this by recording each study's framework and judging comparability case by case rather than restricting eligibility to one set of criteria. This preserves evidence but leaves residual uncertainty about whether the populations are comparable. With four studies, publication bias could not be assessed formally. The search covered publications to 18 May 2026, did not include separate searches of trial registries or reference lists, and required efficacy, safety, or adverse-event terms, which may have missed studies that did not use these terms in the title, abstract, or keywords; relevant studies published after the search date are not included.

Implications for Research

Future trials should prespecify the treatment effect being targeted and the analysis population, use blinded assessment wherever feasible, define clinically meaningful non-inferiority margins prospectively, report both intention-to-treat and per-protocol analyses with confidence limits for non-inferiority designs, and distinguish JAK inhibitor monotherapy from add-on therapy. Consistent PMR outcome definitions and glucocorticoid protocols would improve comparability. Adequate sample sizes and long-term follow-up are essential for safety outcomes.

## Conclusions

Low-certainty evidence from a single small placebo-controlled trial suggests that baricitinib may increase the likelihood of achieving low disease activity without oral glucocorticoids in recent-onset, glucocorticoid-naive PMR. Evidence for tofacitinib comes from one open-label trial whose non-inferiority conclusion is not robust (low certainty) and from observational and single-arm studies (very low certainty). These findings are preliminary and do not establish efficacy for JAK inhibitors as a class. Certainty of evidence is low or very low because of small samples, risk of bias, imprecision, heterogeneous comparators and treatment strategies, and limited safety follow-up. The evidence does not establish a preferred patient population or justify a routine clinical recommendation. Larger, adequately powered, blinded randomised trials with consistent outcome definitions, transparent non-inferiority methods where applicable, and sufficient follow-up for safety are needed.

## Appendices

Appendix A

PRISMA 2020 Checklist

Completed according to the PRISMA 2020 statement [10]. Locations refer to the headings of this manuscript.

**Table 7:** PRISMA 2020 Checklist PRISMA: Preferred Reporting Items for Systematic Reviews and Meta-Analyses; RoB 2: Cochrane Risk of Bias 2 tool; ROBINS-I: Risk Of Bias In Non-randomised Studies of Interventions; NHLBI: National Heart, Lung, and Blood Institute; RR: relative risk; CI: confidence interval; SWiM: Synthesis Without Meta-analysis; GRADE: Grading of Recommendations Assessment, Development and Evaluation; DISS: difficulty in squatting and standing up; PROSPERO: International Prospective Register of Systematic Reviews; JAK: Janus kinase; I²: heterogeneity statistic

Item #	Checklist item	Location in manuscript	Notes
1	TITLE - Identify the report as a systematic review.	Title	
2	ABSTRACT - See the PRISMA 2020 for Abstracts checklist.	Abstract	PRISMA for Abstracts recommends a structured summary, but Cureus permits abstract subheadings only in original articles. The unstructured abstract covers all PRISMA for Abstracts elements in prose.
3	INTRODUCTION: Rationale - Describe the rationale in the context of existing knowledge.	Introduction and Background, paragraphs 1-3	
4	INTRODUCTION: Objectives - Explicit statement of objectives/questions addressed.	Introduction and Background, final paragraph	
5	METHODS: Eligibility criteria - Criteria for inclusion/exclusion and how studies were grouped for synthesis.	Review: Methods, Eligibility Criteria	
6	METHODS: Information sources - All databases/registers/other sources searched, with dates last searched.	Review: Methods, Information Sources and Search Strategy	Search date (18 May 2026) reported for all four databases. Trial registries and reference lists were not searched separately (see Discussion, Limitations).
7	METHODS: Search strategy - Full search strategies for all databases, including filters/limits.	Review: Methods, Information Sources and Search Strategy (summary); Appendix 1 (full strategies)	All four databases used the same three-block structure (population AND intervention AND outcome), the same 11 named agents, and no comparator terms.
8	METHODS: Selection process - Methods to decide study eligibility, number of reviewers, independence, automation tools.	Review: Methods, Study Selection and Data Collection	Two independent reviewers, third-reviewer arbitration; no automation tools used.
9	METHODS: Data collection process - Methods for data extraction, number of reviewers, independence.	Review: Methods, Study Selection and Data Collection	Two independent reviewers, standardised piloted form.
10a	METHODS: Data items - Outcomes sought, definitions, methods to select results.	Review: Methods, Effect Measures and Synthesis	
10b	METHODS: Data items - Other variables collected (e.g. participant/intervention characteristics, funding sources).	Review: Methods, Study Selection and Data Collection	Extraction form covered design, population, intervention, comparator, denominators, risk-of-bias judgements, and safety outcomes (adverse events, serious adverse events, discontinuations, follow-up).
11	METHODS: Study risk of bias assessment - Methods, number of reviewers, independence, tools used.	Review: Methods, Risk of Bias	RoB 2, ROBINS-I, or NHLBI tool matched to study design; second-reviewer check.
12	METHODS: Effect measures - Effect measure(s) used for each outcome.	Review: Methods, Effect Measures and Synthesis	RR for BACHELOR [7]; non-inferiority comparison (not converted to a superiority estimate) for EAST PMR [8]; proportions with 95% CI for the single-arm study.
13a	METHODS: Synthesis methods - Process for deciding eligibility of study results for each synthesis.	Review: Methods, Effect Measures and Synthesis	Synthesis organised by clinically coherent comparison, not pooled.
13b	METHODS: Synthesis methods - Methods for preparing data for presentation/synthesis.	Review: Methods, Study Selection and Data Collection; Effect Measures and Synthesis	
13c	METHODS: Synthesis methods - Methods for tabulating/visually displaying results.	Review: Results, Characteristics of Included Studies (Table 1); Safety (Table 5); Certainty of Evidence (Table 6)	
13d	METHODS: Synthesis methods - Methods used to synthesise results, and rationale.	Review: Methods, Effect Measures and Synthesis	Meta-analysis not performed; rationale given (differences in agent, dose, comparator, outcome definition, design).
13e	METHODS: Synthesis methods - Methods to explore heterogeneity/variation in effects.	Review: Methods, Effect Measures and Synthesis; Review: Discussion, Principal Findings; Interpretation of Glucocorticoid-Sparing Efficacy	Heterogeneity addressed narratively by organising around distinct clinical comparisons rather than statistically (e.g. I²), consistent with SWiM.
13f	METHODS: Synthesis methods - Sensitivity analyses to assess robustness.	Review: Methods, Effect Measures and Synthesis; Review: Results, JAK Inhibitor Versus Glucocorticoid	No pooled analyses; for EAST PMR, the non-inferiority confidence interval was recalculated under alternative assumptions about missing outcome data.
14	METHODS: Reporting bias assessment - Methods to assess risk of bias from missing results.	Review: Methods, Certainty of Evidence	Funnel plots and Egger's test were stated to be uninformative given only four studies.
15	METHODS: Certainty assessment - Methods to assess certainty in the body of evidence.	Review: Methods, Certainty of Evidence	GRADE, applied per clinically coherent comparison rather than per composite outcome.
16a	RESULTS: Study selection - Numbers screened, assessed for eligibility, included, with a flow diagram.	Review: Results, Study Selection; Figure 1	
16b	RESULTS: Study selection - Studies excluded, with reasons, for each study that might appear to meet inclusion criteria.	Review: Results, Study Selection; Figure 1	One report excluded at full text (DISS score companion paper [20]); Camellino et al. [14] retained as contextual evidence rather than excluded.
17	RESULTS: Study characteristics - Citation and characteristics of each included study.	Review: Results, Characteristics of Included Studies (Table 1)	
18	RESULTS: Risk of bias in studies - Risk-of-bias judgements for each study.	Review: Results, Risk of Bias (Tables 2-4)	
19	RESULTS: Results of individual studies - Summary statistics for each study and effect estimate, ideally with a forest plot.	Review: Results, JAK Inhibitor Versus Placebo through Safety	No forest plot; not appropriate because no pooling was performed (see Methods, Effect Measures and Synthesis).
20a	RESULTS: Results of syntheses - Summary of characteristics/risk of bias within each synthesis.	Review: Results, Risk of Bias through Safety	
20b	RESULTS: Results of syntheses - Results of all statistical syntheses, including CIs and heterogeneity measures.	Review: Results, JAK Inhibitor Versus Placebo through Safety	No statistical pooling performed; individual-study effect estimates reported per comparison instead (see Methods, Effect Measures and Synthesis).
20c	RESULTS: Results of syntheses - Results of investigations of heterogeneity.	Review: Discussion, Principal Findings; Interpretation of Glucocorticoid-Sparing Efficacy	Addressed narratively rather than statistically; see item 13e.
20d	RESULTS: Results of syntheses - Results of sensitivity analyses.	Review: Results, JAK Inhibitor Versus Glucocorticoid	See item 13f.
21	RESULTS: Reporting biases - Assessment of risk of bias from missing results for each synthesis.	Review: Methods, Certainty of Evidence; Review: Discussion, Limitations	Formal assessment not performed (too few studies); stated as a limitation.
22	RESULTS: Certainty of evidence - Assessment of certainty for each outcome.	Review: Results, Certainty of Evidence (Table 6)	
23a	DISCUSSION: Interpretation - General interpretation in context of other evidence.	Review: Discussion, Principal Findings; Interpretation of Glucocorticoid-Sparing Efficacy; Clinical Interpretation and Patient Selection; Comparison With Other Targeted Therapies	
23b	DISCUSSION: Limitations of included studies.	Review: Discussion, Limitations	
23c	DISCUSSION: Limitations of the review process.	Review: Discussion, Limitations	
23d	DISCUSSION: Implications for practice, policy, and future research.	Review: Discussion, Clinical Interpretation and Patient Selection; Safety; Implications for Research; Conclusions	
24a	OTHER: Registration - Registration name and number.	Review: Methods, Protocol and Reporting; Abstract	PROSPERO CRD420261364002.
24b	OTHER: Registration - Where the review protocol can be accessed, or a statement that none was prepared.	Review: Methods, Protocol and Reporting	No protocol was prepared beyond the PROSPERO registration record (CRD420261364002).
24c	OTHER: Registration - Amendments to information provided at registration or in protocol.	Review: Methods, Protocol and Reporting; Effect Measures and Synthesis	The registration planned a meta-analysis where appropriate. None was performed because the four studies differed materially in JAK inhibitor, dose, concomitant glucocorticoid exposure, comparator, outcome definition, follow-up, and design, so a pooled estimate would not have been clinically meaningful. Findings were instead synthesised narratively by clinical comparison following SWiM. Recalculation of effect estimates and confidence intervals for BACHELOR and EAST PMR was performed post hoc.
25	OTHER: Support - Sources of financial or non-financial support, and role of funders.	Additional Information: Disclosures (Payment/services info)	No financial support was received for the submitted work.
26	OTHER: Competing interests - Competing interests of review authors.	Additional Information: Disclosures (Conflicts of interest)	All authors declared no conflicts of interest.
27	OTHER: Availability of data, code, and other materials - Availability of data extraction forms, analysis code, and other materials.	Additional Information: Data availability	Data are available in the cited published articles; the completed data extraction form is available from the corresponding author on reasonable request.

Appendix B

Database-Specific Search Strategies

Four databases were searched from inception to 18 May 2026, without language or publication-date restrictions: Embase (Ovid), MEDLINE (Ovid), the Cochrane Library (CENTRAL and Cochrane Reviews), and PubMed. Each search combined three concepts: Population (polymyalgia rheumatica), Intervention (JAK inhibitors, both as a drug class and by 11 named agents), and Outcome (efficacy, safety, adverse events). Comparator terms were not used in any database. Square brackets in the original PubMed and Cochrane syntax (field tags and MeSH descriptors) have been replaced with parentheses to conform to journal style.

MEDLINE (Ovid)

1. polymyalgia rheumatica/ OR polymyalgia rheumatica.ti,ab,kw. OR PMR.ti,ab,kw.

2. Janus kinase inhibitors/ OR (janus kinase inhibitor* OR JAK inhibitor* OR JAKi OR baricitinib OR tofacitinib OR upadacitinib OR ruxolitinib OR filgotinib OR peficitinib OR abrocitinib OR deucravacitinib OR itacitinib OR pacritinib OR fedratinib).ti,ab,kw.

3. (efficacy OR safety OR (adverse adj event*)).ti,ab,kw.

4. 1 AND 2 AND 3

Embase (Ovid)

1. polymyalgia rheumatica/ OR polymyalgia rheumatica.ti,ab,kw. OR PMR.ti,ab,kw.

2. janus kinase inhibitor/ OR (janus kinase inhibitor* OR JAK inhibitor* OR JAKi).ti,ab,kw. OR (baricitinib/ OR tofacitinib/ OR upadacitinib/ OR ruxolitinib/ OR filgotinib/ OR peficitinib/ OR abrocitinib/ OR deucravacitinib/ OR itacitinib/ OR pacritinib/ OR fedratinib/) OR (baricitinib OR tofacitinib OR upadacitinib OR ruxolitinib OR filgotinib OR peficitinib OR abrocitinib OR deucravacitinib OR itacitinib OR pacritinib OR fedratinib).ti,ab,kw.

3. (efficacy OR safety OR (adverse adj event*)).ti,ab,kw.

4. 1 AND 2 AND 3

Cochrane Library (CENTRAL and Cochrane Reviews)

Run: 18/05/2026 02:50:26

#1 MeSH descriptor: (Polymyalgia Rheumatica) explode all trees - 128 hits

#2 (“polymyalgia rheumatica”):ti,ab,kw OR (PMR):ti,ab,kw (word variations searched) - 1,025 hits

#3 PMR:ti,ab,kw - 888 hits

#4 #1 OR #2 OR #3 - 1,025 hits

#5 MeSH descriptor: (Janus Kinase Inhibitors) explode all trees - 280 hits

#6 (Janus Kinase Inhibit*):ti,ab,kw OR (JAK inhibit*):ti,ab,kw (word variations searched) - 1,762 hits

#7 (Tofacitinib):ti,ab,kw (word variations searched) - 1,378 hits

#8 (Ruxolitinib):ti,ab,kw (word variations searched) - 1,055 hits

#9 (Filgotinib):ti,ab,kw (word variations searched) - 424 hits

#10 (baricitinib):ti,ab,kw (word variations searched) - 852 hits

#11 (upadacitinib):ti,ab,kw (word variations searched) - 1,022 hits

#12 (peficitinib):ti,ab,kw - 34 hits

#13 (abrocitinib):ti,ab,kw - 188 hits

#14 (deucravacitinib):ti,ab,kw - 241 hits

#15 (itacitinib):ti,ab,kw - 48 hits

#16 (pacritinib):ti,ab,kw - 99 hits

#17 (fedratinib):ti,ab,kw - 79 hits

#18 #5 OR #6 OR #7 OR #8 OR #9 OR #10 OR #11 OR #12 OR #13 OR #14 OR #15 OR #16 OR #17 - 5,640 hits

#19 (efficacy):ti,ab,kw (word variations searched) - 522,080 hits

#20 (safety):ti,ab,kw (word variations searched) - 355,433 hits

#21 (adverse NEXT event*):ti,ab,kw (word variations searched) - 180,053 hits

#22 #19 OR #20 OR #21 - 706,202 hits

#23 #4 AND #18 AND #22 - 21 hits (final Cochrane result set)

PubMed

(“polymyalgia rheumatica”(MeSH) OR “polymyalgia rheumatica”(tiab) OR “PMR”(tiab)) AND (“Janus Kinase Inhibitors”(MeSH) OR “janus kinase inhibitor*”(tiab) OR “JAK inhibitor*”(tiab) OR “JAKi”(tiab) OR “baricitinib”(tiab) OR “tofacitinib”(tiab) OR “upadacitinib”(tiab) OR “ruxolitinib”(tiab) OR “filgotinib”(tiab) OR “peficitinib”(tiab) OR “abrocitinib”(tiab) OR “deucravacitinib”(tiab) OR “itacitinib”(tiab) OR “pacritinib”(tiab) OR “fedratinib”(tiab)) AND (efficacy(tiab) OR safety(tiab) OR “adverse event*”(tiab))
